# Leukocyte Activation and Antioxidative Defense Are Interrelated and Moderately Modified by *n-3* Polyunsaturated Fatty Acid-Enriched Eggs Consumption—Double-Blind Controlled Randomized Clinical Study

**DOI:** 10.3390/nu12103122

**Published:** 2020-10-13

**Authors:** Martina Mihalj, Ana Stupin, Nikolina Kolobarić, Ivana Tartaro Bujak, Anita Matić, Zlata Kralik, Ivana Jukić, Marko Stupin, Ines Drenjančević

**Affiliations:** 1 Department of Physiology and Immunology, Faculty of Medicine, Josip Juraj Strossmayer University of Osijek, J. Huttlera 4, HR-31000 Osijek, Croatia; martina.mihalj@gmail.com (M.M.); ana.stupin@mefos.hr (A.S.); nikolina.bilic.dujmusic@gmail.com (N.K.); anita.matic@mefos.hr (A.M.); ivana.jukic@mefos.hr (I.J.); marko.stupin@gmail.com (M.S.); 2 Scientific Center of Excellence for Personalized Health Care, Josip Juraj Strossmayer University of Osijek, Trg Svetog Trojstva 3, HR-31000 Osijek, Croatia; 3 Department of Dermatology and Venereology, Osijek University Hospital, J. Huttlera 4, HR-31000 Osijek, Croatia; 4 Department of Pathophysiology, Physiology and Immunology, Faculty of Dental Medicine and Health Josip Juraj Strossmayer University of Osijek, Cara Hadrijana 10E, HR-31000 Osijek, Croatia; 5 Radiation Chemistry and Dosimetry Laboratory, Division of Materials Chemistry, IRB, 10000 Zagreb, Croatia; itartaro@irb.hr; 6 Department of Animal Production and Biotechnology, Faculty of Agrobiotechnical Sciences, Vladimira Preloga 1, HR-31000 Osijek, Croatia; zlata.kralik@fazos.hr; 7 Department for Cardiovascular Disease, Osijek University Hospital, J. Huttlera 4, HR-31000 Osijek, Croatia

**Keywords:** inflammation, microvascular, oxidative stress, *n-3* polyunsaturated fatty acids, endothelium, omega-3 polyunsaturated fatty acids

## Abstract

This placebo-controlled, double-blind, randomized, interventional study investigated the effects of low/intermediate doses of *n-3* polyunsaturated fatty acids (PUFAs) on the endothelial function, markers of leukocyte activation, and oxidative status following dietary intake of *n-3* PUFA-enriched hen eggs in young healthy individuals. Twenty young healthy adults of both sexes who consumed *n-3* PUFA-enriched hen eggs (two eggs per day, for three weeks, total of approximately 407 mg/day *n-3* PUFAs) or regular eggs (two eggs per day for three weeks, total of approximately 75 mg/day *n-3* PUFAs) participated in this study. Skin microvascular endothelium-independent and endothelium-dependent vasodilation were assessed by laser Doppler flowmetry. Serum lipid profile and content of free fatty acids, markers of leukocyte activation, biochemical parameters of oxidative stress, as well as antioxidative enzymes serum activity were measured before and after respective dietary protocol. The results of this study revealed significant differences in the markers of leukocyte activation (such as CD11a/LFA-1) and antioxidative defense, which are related to increased intake of *n-3* PUFAs, providing the evidence that consumption of nutritionally enriched hen eggs may affect physiological processes related to oxidative balance. The absence of significant changes in microvascular reactivity following supplementation with a low-intermediate dose of *n-3* PUFAs, unlike in our previous studies where functional eggs contained ~1 g of *n-3* PUFA, suggests the existence of a dose-dependent effect.

## 1. Introduction

Evidence from multiple experimental, epidemiological, and clinical studies indicate that *n-3* polyunsaturated fatty acids (*n-3* PUFAs) may exert their cardiovascular (CV) protective effect, at least in part, by improving vascular endothelium-dependent function [1,2,3]. These changes occur by *n-3* PUFAs modulation of synthesis of endothelium-derived vasoactive mediators (e.g., NO, COX, and/or CYP450 metabolites), modulation of oxidative stress level, endothelial activation, endothelium-leukocyte interaction, and subsequent endothelial inflammation [1,3,4,5,6]. Previously, we demonstrated the significant beneficiary effect of consumption of *n-3* PUFA-enriched hen eggs on microvascular endothelium-dependent reactivity, even in young healthy individuals [7]. These beneficiary effects were possibly underlined by modulation of pro-inflammatory and anti-inflammatory factors that can influence microvascular reactivity and are produced from *n-3* PUFAs [7].

By changing membrane composition and fluidity directly via specific receptors, saturated and unsaturated fatty acids may influence the regulatory and effector functions of innate and adaptive immune cells [8]. It is well-documented that *n-3* PUFAs have immune-modulatory effects in innate immunity, particularly macrophages. *n-3* PUFAs are able to blunt M1 macrophage polarization upon lipopolysaccharide (LPS) stimulation and to promote M2 polarization [9]. Additionally, *n-3* PUFAs alter secretion of pro-inflammatory and anti-inflammatory cytokines, such as TNF-α, IL-1β, and IL-6, while increasing IL-10 secretion in raw and THP-1-derived macrophages cell lines, as well as in culture of primary cell macrophages (as reviewed in [9]). However, both *n-6* or *n-3* PUFAs are sensitive to oxidative stress and easily peroxided [10]. Oxidative stress influences the activity of enzymes that metabolize *n-3* and *n-6* PUFAs and may modify production of pro-inflammatory or anti-inflammatory metabolites [11]. On the other hand, *n-3* PUFAs metabolites may decrease ROS and NO production in LPS-stimulated macrophages [12], associated with decreased activation of transcription factors involved in the pro-inflammatory responses.

One of the unresolved issue with *n-3* PUFAs consumption is the threshold dose necessary to be consumed to observe these vascular and immune-modulatory effects. The majority of previous studies utilized daily doses above one gram of *n-3* PUFAs in durations from several weeks to several months. For example, in patients with increased CV risk or existing CV disease, these consumed amounts were 1 g to 3.7 g of EPA and/or DHA for 6 to 17 weeks [13,14,15,16]. In our previous studies, consumed doses of *n-3* PUFAs in enriched eggs were 770 and 1051 mg/day for 21 days, respectively [7,17]. Since such high intake of eggs or supplements is not likely in real-life circumstances, the present study aimed to establish the threshold dose for endothelial function modulation and to assess the markers of leukocyte activation and oxidative status (such as biochemical parameters of lipid peroxidation and anti-oxidative enzymes activity) in a randomized, double-blind, controlled prospective study in young healthy participants of both sexes.

## 2. Materials and Methods

### 2.1. Participants and Study Design

This was a randomized, double-blind, placebo-controlled interventional study (registration on ClinicalTrials.gov Identifier: NCT02720250). Twenty young healthy individuals were recruited to participate in this study, including 9 women and 11 men. All volunteers were initially examined to determine if they met the inclusion criteria, including (a) age of subjects ranging between 18 and 30 years, (b) body mass index (BMI) within the normal range (18.5–24.9), as well as normal values of (c) arterial BP and (d) serum lipids. Individuals with a positive history of smoking, hypertension, coronary artery disease, diabetes, hyperlipidemia, renal impairment, cerebrovascular and peripheral artery disease, or taking any drugs or substances that could affect the endothelium were excluded from the study. Study protocol consisted of a dietary intervention during the period of three weeks (21 day) in which the subjects were instructed to eat two hen eggs per day (total of 42 eggs provided by the investigators), and of two study visits (on day 1 and 21) during which blood sampling and functional measurements were performed (described in the following Section 2.3, Section 2.4, Section 2.5, Section 2.6, Section 2.7, Section 2.8, Section 2.9, Section 2.10). Participants were randomly assigned to one of the two study groups: (1) *n-3* PUFA group (*n* = 11) in which subjects received *n-3* PUFA-enriched hen eggs (approximately 407 mg of *n-3* PUFAs per day), or control group (*n* = 9) in which the subjects were provided with regular hen eggs (approximately 75 mg of *n-3* PUFAs per day). Subjects were instructed to eat eggs boiled for about 10 min prior to consumption, and were asked not to take additional eggs or *n-3* PUFAs supplementation in any form prior to enrolment or during when the study protocol study was performed in the Laboratory for Clinical and Sport Physiology, Department of Physiology and Immunology at Faculty of Medicine, University of Osijek. All participants signed a written informed consent. The study protocol and procedures conformed with the standards set by the latest revision of the Declaration of Helsinki and were approved by the Ethical Committee of the Faculty of Medicine, University of Osijek (Cl: 602-04/14-08/06; No: 2158-610714-114).

### 2.2. Functional Eggs Production and Fatty Acids Content

*n-3* PUFA-enriched hen eggs and the regular eggs were produced and provided by the Faculty of Agrobiotechnical Sciences, University of Osijek, who developed and standardized the production protocols. In short, the common sunflower oil in laying hen feeding mixtures was replaced by a mixture of fish (1.33%), linseed (1.33%), rapeseed (1.33%), and soybean (1%) oil [7]. The composition of fatty acids in the feeding mixture used for the production of regular and *n-3* PUFA-enriched eggs is shown in Table 1. As a result of changing the fatty acid content in the feeding mixture, hen eggs reduced *n-6* PUFAs and increased *n-3* PUFA concentration with more favorable *n-6*/*n-3* PUFAs ratio (Table 1). The fatty acid profile of hens’ feeding mixture and the edible parts of eggs was determined according to the method of Csapo et al. [17], and the procedure was described in detail in our previously published paper [7]. Gas chromatography was performed on a Bruker 430-GC apparatus (Billerica, MA, USA), equipped with a FAMEWAX (RESTEK, Bellefonte, PA, USA) type capillary column (30 m × 0.32 mm internal diameter, 0.25-μm film) and flame ionization detector. To identify individual fatty acids in the chromatogram, a fatty acid standard mixture Supelco 37 Component FAME Mix (Supelco Inc., Bellefonte, PA, USA) was used. Fatty acids profiles of edible parts of regular and *n-3* PUFA-enriched eggs (mg/100 g edible part) are described in Table 1. Each *n-3* PUFA egg (average weight 60 g) contained on average 203 mg of *n-3* PUFAs (ALA 100 mg/egg, EPA 11 mg/egg, DHA 91 mg/egg). Each control egg (average weight 60 g), produced on the same farm, contained on average 38 mg of *n-3* PUFAs (ALA 12 mg/egg, EPA 6 mg/egg, DHA 20 mg/egg).

### 2.3. Anthropometric, Body Composition, Body Fluid Status, and Arterial Blood Pressure Measurement

Subjects’ weight, height, and hip and waist circumference were measured, and waist-to-hip (WHR) ratio and body mass index (BMI) were calculated using appropriate formulas. A four-terminal portable impedance analyzer (Maltron Bioscan 920-II, Maltron International Ltd., Rayleigh, Essex, UK) was used to measure body fluid status and body composition, by which software and empirically derived formulas estimated fat% (fat mass%), fat free mass% (FFM%), extracellular water% (ECW%), intracellular water% (ICW%), total body water% (TBW%), interstitial fluid (IF), plasma fluid (PF), and body density (kg/L) were calculated. Blood pressure (BP) and heart rate (HR) were measured using an automated oscillometric sphygmomanometer (OMRON M3, OMRON Healthcare Inc., Osaka, Japan) in a seated position. The final values of BP and HR were the mean of three repeated measurements.

### 2.4. Assessment of Forearm Skin Microvascular Reactivity

Forearm skin post-occlusive reactive hyperaemia (PORH), which is a microvascular response to vascular occlusion and microvascular reactivity response to iontophoresis of acetylcholine (ACh) (endothelium-dependent vasodilation) and sodium nitroprusside (SNP) (endothelium-independent vasodilation), were measured by laser Doppler flowmetry (LDF) (MoorVMS-LDF, Axminster, UK). Microcirculatory blood flow was expressed in arbitrary perfusion units and determined by software calculating the area under the curve (AUC). The final result for the PORH test was expressed as the difference between percentage of flow change during reperfusion and occlusion in relation with baseline (R-O%), and for SNP or ACh iontophoresis test as a blood flow increase following SNP or ACh administration in relation to baseline flow (SNP or ACh blood flow increase). LDF measurements were performed in a temperature-controlled room. General procedures for performing LDF PORH test and LDF iontophoresis of ACh and SNP tests were done in accordance with our well-established protocols, which are described in detail in previous papers of our research group [18,19,20].

### 2.5. Venous Blood Biochemical Analysis

A venous blood samples for the analysis described in this and further Section 2.5, Section 2.6, Section 2.7, Section 2.8, Section 2.9 and Section 2.10 were taken after 15 min resting in a seated position at each visit. Blood samples were analyzed for full blood count, plasma electrolytes (calcium, sodium, potassium), urea, creatinine, urates, transferrin, iron, fasting lipid panels [total cholesterol, triglycerides, low-density lipoprotein cholesterol (LDL), and high-density lipoprotein cholesterol (HDL)], apolipoprotein A (apoA) and apolipoprotein B (apoB), high sensitivity C reactive protein (hsCRP), and fasting blood glucose using standard laboratory methods. Described analyses were performed at the Department of Clinical Laboratory Diagnostics, University Hospital Osijek.

### 2.6. Profiling of Plasma Fatty Acid Concentrations

Profiling of plasma fatty acids concentrations was done in the Radiation Chemistry and Dosimetry Laboratory, Division of Materials Chemistry, Ruđer Bošković Institute (IRB), Zagreb, Croatia, according to previously described protocol [21]. Total lipids were extracted from plasma and treated with 0.5 M KOH/MeOH (Merck, Germany) to induce formation of the fatty acid methyl esters (FAMEs). Gas chromatography was used for FAMEs extraction and analysis. Individual fatty acids were determined in plasma samples and expressed as relative percentages of FAME obtained from total lipid extract. The percentage of FAME (a relative amount of each fatty acid) was quantified by integrating the area under the peak and dividing the results by the total area for all fatty acids [21].

### 2.7. Measurement of Thiobarbituric Acid Reactive Substances (TBARS) and Ferric-Reducing Ability of Plasma (FRAP)

Spectrophotometry measurement of the thiobarbituric acid reactive substances (TBARS), which are products of lipid peroxidation and presents the measure of oxidative stress, and the ferric-reducing ability of plasma (FRAP), which presents the measure of antioxidant capacity, were done in all participants before and after respective diet protocol according to the previously described protocol from our laboratory [22,23].

### 2.8. Measurement of Glutathione Peroxidase 1 (GPx1) and Superoxide Dismutase (SOD) Serum Activity

As a measure of antioxidant potential, the serum activity of antioxidant enzymes superoxide dismutase (SOD) and glutathione peroxidase (GPx) were measured before and after respective diet protocol using previously established methods by our research group [22,24,25]. GPx activity was determined indirectly by measuring the rate of nicotinamide adenine dinucleotide phosphate (NADPH) oxidation to NADP+, accompanied by a decrease in absorbance at 340 nm over 5 min. Total SOD activity was measured as the degree of inhibition of reduction of cytochrome C by a superoxide radical. Enzyme activity assays were performed using a Lambda 25UV-Vis spectrophotometer equipped with UV WinLab 6.0 software package (PerkinElmer For the Better, Waltham, MA, USA). Measured activities of all investigated enzymes were expressed as U (mg protein)-1.

### 2.9. Measurement of Plasma Lipid Hydroperoxides (LOOH) Concentration

Plasma lipid hydroperoxides (LOOH) presents another biochemical marker of oxidative stress level. Lipids extraction in plasma samples was performed as previously described [26]. The concentrations of LOOH were calculated using the molar absorptivity of the complex [FeNCS]^2+^ formed per mol of LOOH, 58,440 M^−1^ cm^−1^, at 500 nm [27,28]. Measurements were performed in the Laboratory for Radiation Chemistry and Dosimetry, Ruđer Bošković Institute, Zagreb, Croatia.

### 2.10. Flow Cytometry

In order to assess activation status of peripheral blood leukocyte subpopulations, cell surface expression of LFA-1 (CD11a/CD18) integrin, as well as frequencies of CD11a baring leukocyte subsets were determined by flow cytometry. Venous blood samples were collected in tubes containing EDTA at concentration of 1.8 mg EDTA per milliliter of blood, and immediately processed for cell-surface staining. One-hundred μL of whole blood was stained and incubated with a mixture of antibodies (50 μL per sample) for 30 min at room temperature in the dark. Prior to the addition of antibody mixture, nonspecific binding was blocked by addition of 5 uL Fc block reagent and 10 min of incubation at room temperature (Human BD Fc Block, BD Pharmingen, San Jose, CA, USA). The antibody mixture consisted of CD14 FITC (clone: 61D3, eBioscience, San Diego, CA, USA), CD45-PerCP (clone: MEM-28, EXBIO, Vestec, Czech Republic), biotinylated CD11a/LFA-1 (clone: HI111, eBioscience, San Diego, CA, USA), and CD16 APC (clone: eBioCB16, eBioscience, San Diego, CA, USA. Visualization of the biotinylated antibody was performed by an additional staining step with streptavidin coupled with 100 μL PE-Cy7 (BD Biosciences, San Jose, CA, USA) on room temperature for 30 min in the dark. Erythrocytes were lysed by cell incubation with 1x BD™ FACS™ Lysing Solution for 10 min on 37 °C. After the last washing step with 1 × PBS, cells were fixated with 1% formaldehyde. At least 10,000 target cells were collected by a BD FACSCanto II cytometer (FACSCanto II, Becton Dickinson, San Jose, CA, USA) and analyzed using the FlowLogic software (Inivai Technologies, Mentone, Australia).

### 2.11. Statistical Analysis

All results are reported as the arithmetic mean ± standard deviation (SD). The Kolmogorov–Smirnov normality test was used to assess the distribution of the acquired data. Within group analysis was performed using the Wilcoxon rank-sum test in the case when variables were not normally distributed or a paired student’s *t*-test for normally distributed data. All results were additionally compared by one-way ANOVA or Kruskal–Wallis test, followed by Holm–Sidak or Dunn’s post hoc test, respectively. The correlations between functional data or biochemical blood analysis data and corresponding immunological parameters were determined by Spearman’s or Pearson’s correlation tests when appropriate. *p* < 0.05 was considered statistically significant. For statistical. analysis the SigmaPlot, version 11.2 (Systat Software, Inc., Chicago, IL, USA) was used.

## 3. Results

### 3.1. Patients Characteristics, Hemodynamic and Biochemical Parameters

All participants completed a 3-week dietary protocol. All participants were normotensive and lean, had normal renal function, full blood count, serum electrolytes, iron, transferrin, hsCRP, fasting blood glucose, and fasting lipid levels (Table 2). Age, anthropometric, and hemodynamic parameters (e.g., age, BMI, WHR, BP and HR) were not significantly different at the moment of entering the study protocol between participants who comprised control and *n-3* PUFAs group (Table 2 and Table 3). Sodium and HDL cholesterol were significantly higher, and triglycerides significantly lower in *n-3* PUFAs group compared to control group at the moment of entering the study protocol (Table 2); however, all values were within normal population range. Values of other biochemical parameters at the moment of entering the study protocol between the participants in control and *n-3* PUFAs groups were similar (Table 2).

BP, HR, and biochemical values in control and *n-3* PUFAs group before and after respective protocol are presented in Table 2. BP and HR were similar after dietary protocol compared to baseline (initial) measurements within *n-3* PUFAs or control group. Also, the values of SBP, MAP, DBP, and HR were similar between control and *n-3* PUFAs group following corresponding diet protocol.

Hemoglobin and hematocrit values were significantly increased after dietary intake of regular hen eggs compared to initial measurement in control group. Thrombocyte level significantly increased, and fasting blood glucose significantly decreased at the end of dietary protocol compared to initial measurement in *n-3* PUFAs group. Transferrin level significantly increased in both control and *n-3* PUFAs group following respective diet protocol compared to baseline measurement. However, all values (hemoglobin, hematocrit, thrombocyte, and transferrin) were within physiological range. There were no significant differences in erythrocytes and leukocytes number, as well as serum urea, creatinine, urates, electrolytes, iron, hsCRP, and fasting lipid panel concentrations after *n-3* PUFAs enriched or regular eggs consumption compared to baseline conditions within the groups, or compared between the groups.

### 3.2. Anthropometric Measures, Body Fluid Status, and Body Composition

BMI, WHR, body fluid status, and body composition in control and *n-3* PUFAs group before and after respective protocol are described in Table 3. Despite randomization in assignment to groups, at baseline, control group had significantly higher FFM% and body density compared to *n-3* PUFAs group. Hence, at baseline *n-3* PUFAs group had significantly higher fat% than controls. This difference in fat% was lost after dietary protocols. WHR, BMI, FFM%, ECW%, ICW%, TBW%, IF, PF, or Body Density were not affected by dietary protocols in both control and *n-3* PUFAs group (Table 3). BMI, WHR, body fluid status, and body composition were similar between control and *n-3* PUFAs group following corresponding diet protocol.

### 3.3. Plasma Fatty Acid Concentrations

Dietary intake of *n-3* PUFA-enriched eggs did not significantly change plasma fatty acids profile compared to baseline measurements in *n-3* PUFAs group (Table 4). Intake of regular eggs significantly decreased plasma concentration of oleic acid (C18:1; *n-9* PUFA) compared to baseline measurement in control group. Also, at baseline measurement, oleic acid plasma concentration was significantly higher in control than in *n-3* PUFAs group. There were no other significant differences in plasma fatty acid concentrations compared within the groups, or compared between the groups (Table 4).

### 3.4. Acetylcholine-Induced Dilation, Sodium Nitroprusside-Induced Dilation, and Post-Occlusive Reactive Hyperaemia in Forearm Skin Microcirculation

Consumption of regular or *n-3* PUFA-enriched eggs did not significantly change PORH, AChID, or SNPID compared to baseline measurement in control and *n-3* PUFAs group, respectively (Supplementary Materials Figure S1). Also, values of PORH, AChID, and SNP were similar between the groups, before and after respective diet protocol (Figure S1).

### 3.5. Markers of Oxidative Stress and Antioxidative Defence

A three-week dietary consumption of *n-3* PUFA-enriched eggs significantly decreased FRAP in *n-3* PUFAs group compared to baseline (Table 5), while FRAP remained unchanged following regular eggs consumption after dietary protocol in the control group. TBARS level was significantly higher in *n-3* PUFAs than in control group at baseline, as well as after completion of respective egg consumption protocol. However, TBARS was not significantly affected by both *n-3* PUFAs or regular egg consumption compared to baseline within the groups.

**Figure 1:**
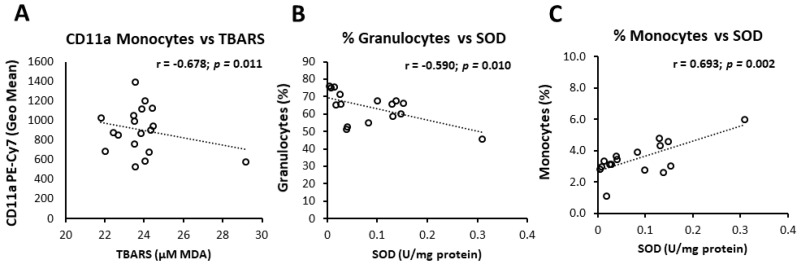
Correlation of oxidative status markers and peripheral blood leukocyte subpopulations or CD11a expression. Levels of lipid peroxidation negatively correlated to CD11a expression monocytes in Control group (**A**), while in the *n-3* PUFA group SOD activity negatively and positively correlated to frequencies of peripheral blood granulocytes (**B**) and monocytes (**C**), respectively. Correlation coefficient (r) was calculated using Pearson product-moment correlation. *p* < 0.05 was considered significant. Control group—young healthy volunteers subjected to 14-day diet with regular hen eggs; *n-3*PUFA group—young healthy volunteers subjected to 14-day diet with *n-3* PUFA-enriched hen eggs. PUFA—Polyunsaturated Fatty Acids, CD11a—Cluster of Differentiation 11a, integral part of integrin Lymphocyte Function-Associated Antigen 1 (LFA-1), TBARS—Thiobarbituric Acid Reactive Substances, MDA—Malondialdehyde, SOD—Superoxide Dismutase.

Consumption of *n-3* PUFAs enriched eggs significantly increased SOD serum activity in *n-3* PUFAs group compared to baseline (Table 5, Figure 1), while it remained unchanged following regular eggs consumption compared to baseline in control group. SOD serum activity was similar between the groups. GPx serum activity was not significantly changed after *n-3* PUFAs or regular egg consumption compared to baseline within the groups, just as GPx serum activity was similar between the groups.

LOOH plasma concentration was not significantly changed by either *n-3* PUFAs or regular egg consumption compared to baseline within the groups, just as LOOH plasma concentration was similar between the groups (Table 5).

### 3.6. Interrelation of Vascular Measurements, Markers of Oxidative Balance, and Leukocyte Activation

Additional analysis revealed significant association of individual oxidative status markers and BMI, frequencies of peripheral blood leukocyte subsets and CD11a/LFA-1 expression levels. Namely, in the control group, a significant negative correlation between TBARS and CD11a/LFA-1 expression on monocytes was found (*r* = −0.678, *p* = 0.011; Figure 1), while in *n-3* PUFAs group, TBARS was increasing together with increasing BMI levels (*r* = 0.462, *p* = 0.039; Table 6). In the *n-3* PUFAs group, SOD activity correlated negatively to frequencies of peripheral blood granulocytes (*r* = −0.590, *p* = 0.001; Figure 1) and BMI (*r* = −0.548, *p* = 0.039; Table 6), while SOD activity and peripheral blood monocyte frequencies correlated positively (*r* = 0.534, *p* = 0.015; Figure 1, Table 6).

In the case of control group, we additionally found a significant positive association between CD11a/LFA-1 expression on granulocytes or monocytes and forearm skin microvascular response to iontophoresis of acetylcholine (*r* = 0.556, *p* = 0.038 or *r* = 0.541, *p* = 0.043; Figure S1).

### 3.7. Peripheral Blood Leukocyte Subpopulations and CD11a (LFA-1) Integrin Expression

**Figure 2:**
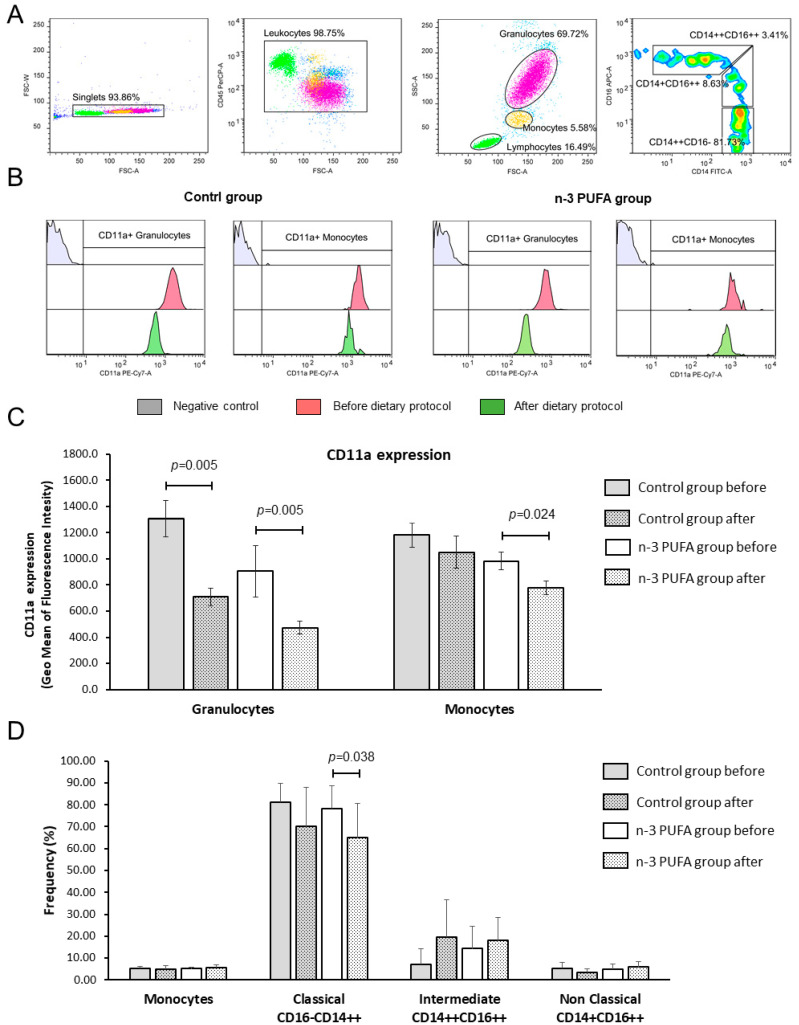
Effects of regular and *n-3* PUFA-enriched eggs consumption on the LFA-1 (CD11a) integrin expression. The gating strategy is shown in panel (**A**). Representative histograms demonstrating CD11a integrin expression on granulocytes and monocytes in negative control samples (grey), at baseline (red), and after respective dietary protocol (green) are shown in panel (**B**). Expression levels of CD11a integrin on granulocytes and monocytes are demonstrated in panel (**C**), while average frequencies of peripheral blood monocytes, classical (CD14++CD16−), intermediate (CD14++CD16++), and non-classical monocytes (CD14+CD16++) are demonstrated in panel (**D**). Data are presented as arithmetic mean and standard deviation; within group differences were tested using the Wilcoxon rank-sum test or paired student’s *t*-test, while between group differences were compared using one-way ANOVA or Mann–Whitney U test, *p* < 0.05 were considered significant. Control group—young healthy volunteers subjected to 14-day diet with regular hen eggs; *n-3* PUFAs group—young healthy volunteers subjected to 14-day diet with *n-3* PUFA-enriched hen eggs. SSC-A—Side scatter, FSC-A—Forward Scatter, CD11a, PUFA—Polyunsaturated Fatty Acids, CD11a—Cluster of Differentiation 11a, integral part of integrin Lymphocyte Function-Associated Antigen 1 (LFA-1), PE-Cy7-A—fluorescence tandem dye consisting of R-phycoerythrin (PE) and Cyanine-7 (Cy7).

**Figure 3:**
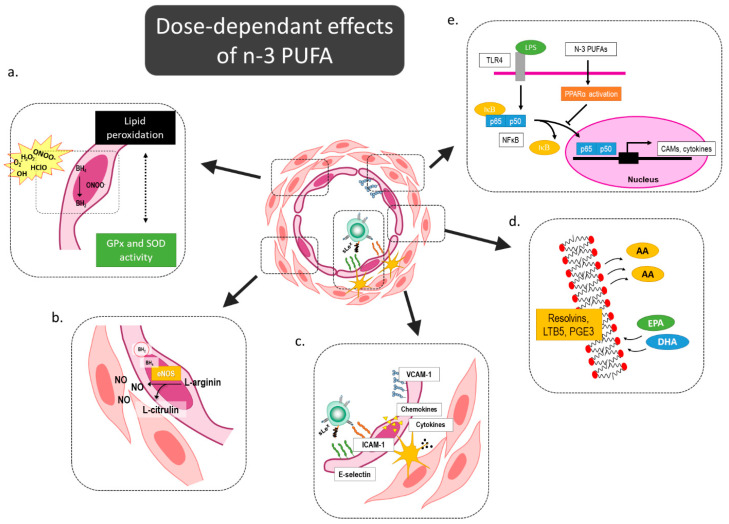
Dose-dependent effects of *n-3* PUFAs on microcirculation. *N-3* PUFAs can modulate oxidative balance by increasing activity of oxidative enzymes and reducing lipid peroxidation (**a**), and thereby prevent e-NOS uncoupling and improve/restore endothelial function in cardiovascular patients (**b**). Several previous functional studies demonstrated reduced endothelium-leukocyte interactions and a subsequent lower level of leukocyte extravasation (**c**). *N-3* PUFAs outcompete *n-6* PUFAs for the integration into the cell membrane, and as a result of these structural changes of the membrane, the products of lipoxygenase and cyclooxygenase pathway are anti-inflammatory mediators, such as resolvins, prostaglandin E3, and leukotriene B5 (**d**). In addition, *n-3* PUFA attenuate endothelial activation trough PPAR activation-mediated NFκB inhibition (**e**). eNOS—endothelial nitric oxide synthase; NFκB—nuclear factor kappa-light-chain-enhancer of activated B cells; PPAR—Peroxisome proliferator-activated receptor PUFA—Polyunsaturated Fatty Acids, GPx—Glutathione Peroxidase; SOD—Superoxide Dismutase; AA—Arachidonic Acid; EPA—Eicosapentaenoic Acid; DHA—Docosahexaenoic Acid.

At the end of the dietary protocols, frequencies of peripheral blood monocytes and granulocytes in both groups remained the same and were similar between the groups. Interestingly, in both groups, frequencies of peripheral blood monocytes negatively correlated to BMI (Figure 2). The rate of CD11a bearing leukocytes among CD45+ blood cells was in the range between 99 and 100%, and the rate of CD11a positive monocytes, granulocytes or lymphocytes did not change significantly upon the dietary protocol (Tables S1 and S2). At the end of dietary protocol, both groups presented with significantly reduced expression of LFA-1/CD11a integrin on the peripheral blood granulocytes (control group 1307.5 ± 392.3 vs. 707.8 ± 197.5; *p* = 0.005; Figure 2C and *n-3* PUFAs group 982.2 ± 227.44 vs. 776.8 ± 177.91; *p* = 0.005; Figure 2C) when compared to the respective baseline levels. In addition, *n-3* PUFAs group exhibited monocytes deactivation, as evidenced by significantly reduced CD11a/LFA-1 expression compared to baseline values (904.4 ± 644.6 vs. 473.0 ± 162.4; *p* = 0.024; Figure 2C). CD11a/LFA-1 expression was similar between the two groups at baseline or after the dietary protocols.

Peripheral blood monocytes were additionally analyzed for classical (CD14++CD16−), intermediate (CD14++CD16++), and non-classical (CD14+CD16++) subsets, and we found significantly reduced frequency of classical monocytes in the *n-3* PUFAs group after the dietary protocol (*p* = 0.039; Figure 2D) with a slight (but not statistically significant) shift towards the intermediate subset.

## 4. Discussion

In the present study, we investigated the effects of a diet enriched with low doses of *n-3* PUFAs on the microvascular function, oxidative status, and antioxidative capacity, as well as the expression of leukocyte inflammation markers in young healthy individuals. Based on our results, daily consumption of two fortified hen eggs (~407 mg *n-3* PUFAs) was insufficient to induce significant changes in serum concentrations of *n-3* and *n-6* PUFAs or the skin microvascular reactivity. However, we found a small but significant immunomodulatory effect of hen eggs consumption independent of *n-3* PUFAs content, in respect of reduced integrin CD11a/LFA-1 expression on the populations of peripheral blood monocyte and granulocyte accompanied by a shift from classical to intermediate monocyte subsets, suggesting a decrease in pro-inflammatory phenotype and deactivation of leukocyte subpopulations. These effects were more pronounced in group receiving *n-3* PUFA-enriched than regular hen eggs.

Surprisingly, although the subjects were recruited among young, healthy, normal weight individuals matched by sex and age, their random assignment to two experimental groups resulted in the significantly higher body fat composition in the *n-3* PUFAs group and discrete changes in oxidative balance, suggesting pro-oxidative state related to the body fat content. This was supported by a significant association between BMI and markers of oxidative balance, which is probably present due to the higher level of lipid peroxidation in those individuals (as evidenced by TBARS levels). Interestingly, consumption of *n-3* PUFA-fortified eggs resulted in compensatory increase in SOD activity in the same group, the effect of which was not observed in control group.

### 4.1. n-3 PUFA Enriched Hen Eggs Effects on Microvascular Functional Responses

In the present study, microvascular reactivity was not significantly affected by any kind of hen eggs consumption. This contrasts with our previous findings of enhanced microvascular dilator responses in young individuals who consumed *n-3* PUFA-enriched hen eggs [7,29]. However, this difference may be contributed to the low dose of ingested *n-3* PUFAs (approximately 406 mg/day for three weeks in the present study, compared to 770 or 1050 mg/day in previous studies). Improved endothelium-dependent vasodilation in response to acetylcholine was reported in healthy individuals who took fish oil supplementation (EPA+DHA) of approximately 250 mg/day for eight months [30]. In an effort to determine cut-off values of *n-3* PUFAs supplementation from which their beneficial effects on endothelial function may begin to emerge, in this particular study we tested if supplementation of 406 mg *n-3* PUFAs daily in a form of functional food would induce functional improvement of forearm skin microvascular function, as evidenced in our earlier study [7,29]. Taken together, the cut-off values of dietary intake of *n-3* PUFAs may be set at >750 mg per day, during at least three weeks in order to observe beneficial functional changes in the microcirculation of young healthy individuals.

### 4.2. n-3 PUFA-Enriched Hen Eggs, Blood Pressure Level, and Serum Lipid Profile

Most studies reporting that *n-3* PUFAs intake has the potential to induce clinically relevant reduction in BP level were done in patients with essential hypertension, in patients with untreated hypertension, and in normotensive but mildly hypercholesterolemic individuals [31,32,33,34]. However, such a significant effect of *n-3* PUFAs intake in a form of fish servings (∼800 mg/serving EPA+DHA; four servings a week for eight weeks) or supplementation as fish oil capsules (two grams/day EPA+DHA for 12 weeks; 1.7 g/day EPA+DHA for four weeks) in lowering BP level was not observed in normotensive individuals [35,36,37]. Interestingly, two studies reported significantly decreased BP level following consumption of *n-3* PUFA-enriched hen eggs in healthy individuals (770 mg/day for three weeks [29,38]; four eggs/day for four weeks). In the present study, consumption of *n-3* PUFA-enriched hen eggs did not significantly affect BP levels.

Furthermore, in the present study no significant changes in serum lipid profile (triglycerides, total cholesterol, LDL cholesterol, and HDL cholesterol) following consumption of *n-3* PUFA-enriched hen eggs in healthy individuals were observed. Clinical studies and meta-analysis in hyperlipidemic individuals reported that *n-3* PUFAs supplementation (EPA and DHA) can reduce serum lipids, most notably triglycerides [1]. Moreover, consumption of *n-3* PUFA-enriched functional food (e.g., hen eggs) has the potential to decrease serum triglycerides level (for approximately 16–18%) in healthy individuals [29,38,39,40]. Altogether, current evidence indicate that *n-3* PUFA intake has potential to decrease serum triglycerides level in both hyperlipidemic and normolipidemic individuals, but obviously, there is a threshold amount of *n-3* PUFAs that needs to be consumed to observe these effects.

### 4.3. Modulation of Oxidative Stress and Antioxidative Capacity by n-3 PUFA-Enriched Diet

Antioxidant enzymes, such as glutathione peroxidase (GPx), superoxide dismutase (SOD), and catalase (CAT), serve as a primary defense system against oxidative stress [41,42,43,44,45,46,47,48]. Although these scavenger enzymes at first are inactivated when counteracting ROS, their expression is induced by the presence of reactive species [41]. Lower levels of serum SOD in hypertensive and diabetic subjects are negatively correlated with most of the vascular functional and structural parameters [49]. Subjects suffering from diabetes and lipid profile impairment exhibit low antioxidant activity of SOD and GPx [50,51].

Our participants were young healthy individuals with no relevant impairments or disease history that could alter antioxidative activity or serum levels of observed enzymes. Serum levels of SOD significantly increased in *n-3* PUFAs group after the protocol, which suggests beneficial effect of *n-3* PUFAs on this defense system. On the other hand, observed increases in SOD activity may be a compensation for a decrease in plasma antioxidative capacity (measured by FRAP) in *n-3* PUFAs group. SOD scavenges oxygen radicals and inhibits lipid peroxidation, thus preventing detrimental effects of oxidative stress in vasculature [52]. This may explain why lipid peroxidation products in plasma of *n-3* PUFAs group were not affected by dietary protocol. In the early phases of lipid peroxidation, as a result of fatty acid oxidation, lipid hydroperoxides (LOOH) are formed. Interestingly, we did not observe changes in LOOH products related to dietary protocol. LOOH are generated from PUFAs and represent primarily products of fatty acid peroxidation, while malondialdehyde presents an end product of lipid peroxidation and is often measured as thiobarbituric acid reactive substances (TBARS) [53]. LOOHs may give epoxy-allylicperoxyl radicals (OLOO·) by undergoing iron-mediated electron reduction and oxygenation [53]. Additional protection from iron-induced oxidative stress is provided by transferrin, which binds extracellular iron [54]. Although within physiological range, transferrin was significantly increased after both dietary protocols in the present study. It is interesting that activation of M1 macrophages is associated with reduction of ferroportin and upregulation of iron storage protein H ferritin, which leads to iron retention and inflammation [55]. In the present study, we observed decreased frequency of classical monocytes but increased transferrin with *n-3* PUFAs dietary intake, which supports the hypothesis that transferrin participates in antioxidative defense and is also employed by *n-3* PUFAs.

In our study, plasma antioxidative capacity was slightly, but significantly decreased in *n-3* PUFAs group after dietary protocol compared to baseline levels. This may be related to unchanged activity of plasma GPx and increased plasma lipid peroxidation end-products at the beginning of the protocol. Furthermore, BMI correlates negatively with SOD and GPx activity in *n-3* PUFAs group. In addition, participants in *n-3* PUFAs group had significantly increased fat%, which positively correlated with TBARS, suggesting that fat body content can be a source of increased oxidative stress in these participants.

### 4.4. Supplementation with Intermediate Doses of n-3 PUFAs Deactivates Circulating Leukocytes at Comparable Levels to Regular Eggs

There is an accumulating amount of evidence that dietary consumption of *n-3* PUFAs can favorably modify immune processes and lower the risk of cardiovascular events and mortality for individuals who consume increased amounts of DHA and EPA [56]. It seems that the immunomodulatory effects of *n-3* PUFAs are more easily observed and traced if the human clinical trials involve subjects with pre-existing conditions [56,57]. One of the most prominent changes observed is the reduction in endothelium-leukocyte interactions, which results in preventing the initiation and slowing the progress of atherosclerosis [57]. *n-3* PUFAs (including EPA and DHA, as well as their oxidized metabolites) can decrease the expression of key adhesion molecules, such as intercellular adhesion molecule (ICAM)-1), vascular cell adhesion molecule (VCAM)-1 or E-Selectin on the surface of endothelial cells (as reviewed in [57]). Integrins (CD11/CD18, VLA-4) on leukocytes interact with immunoglobulin-like adhesion molecules (e.g., ICAM, VCAM-1) on endothelial cells, thus mediating firm adhesion and subsequent trans-endothelial migration of leukocytes [58]. Upon in vitro TNF alpha-induced human umbilical vein endothelial cells activation, preincubation of these cells with EPA or DHA resulted in reduced rolling and adherence of monocytes on activated endothelial cells without affecting CAMs expression. This was further related to the suppressed generation of endothelial platelet-activating factor (PAF) [59]. In the present study, we found evidence for leukocyte deactivation, possible through reduced endothelial activation characterized by downregulation of CAMs and inflammatory mediators. However, in the control group that followed the diet with regular hen eggs, similar effects were also observed. This could be attributed to the under-recognized anti-oxidative properties of hen eggs. Namely, beside their exceptional nutritive value, and overestimated atherogenic effects, hen eggs are very rich in antioxidants like ovalbumin, ovotransferrin, ovomucin, lysozyme, cystatin, carotenoids, vitamin E, etc. [60]. Furthermore, it has been found that lysozyme from hen egg white ameliorates LPS-induced systemic inflammation in mice [61]. Sethi S et al. showed that the treatment with oxidized EPA, but not native EPA, diminished LPS-induced surface expression of E-selectin, VCAM-1, and ICAM-1 in a dose dependent manner, suggesting an important role for oxidized metabolites of *n-3* PUFAs [62]. Furthermore, it was shown that DHA can promote the resolution of inflammation by facilitating efferocytosis through M2 macrophage polarization, and this was linked to *n-3* PUFA-induced PPAR γ activation [63].

Unlike the CD11a/LFA-1 expression on leukocytes that was observed in both groups consuming regular or *n-3* PUFA-fortified hen eggs, in the case of monocyte subsets distribution, there was *n-3* PUFA-related decrease in classical CD14++CD16—monocytes accompanied by a shift towards intermediate CD14++CD16++ monocytes. This is in line with a study on lipoprotein receptor deficient mice (LDLr−/−) fed a diet rich in fish oil, which exhibited significantly reduced plasma cholesterol levels, frequency of splenic Ly6Chi (classical) monocytes, atherosclerosis, monocyte trafficking into the aortic root, and atherosclerotic lesion macrophage content [64]. Similar research performed on humans with hypertriglyceridemia showed reduced fasting triglyceride levels and decreased proportions of intermediate monocytes [65]. Arnardottir HH et al. reported that the proportion of classical monocytes in the blood of healthy mice was decreased upon dietary fish oil consumption, but their proportion was increased upon induction of inflammation, thus enhancing microbial defense [66].

Oxidative stress may promote monocyte recruitment in human diseases. For example, H_2_O_2_ was shown to serve as chemoattractant to monocytes [67], while peroxynitrate enhances expression of VCAM, P-selectin, and E-selectin on endothelial cells in a process possibly mediated by heme oxygenase-1 (HO-1) regulation [68]. Vice versa, activated endothelial cells produce ROS, which by activating oxidant-sensitive transcription factors (AP-1, NFkB), promotes transcription of CAMs [69]. A decrease in oxidative stress and intracellular ROS level of human monocytes, reduced their migration and adhesion [70]. In the present study, positive association between SOD activity and the frequency of peripheral blood monocytes was found. This would suggest that the increased SOD activity may compensate for increased inflammation, e.g., decreased monocyte activation and extravasation. One interesting finding of this study was the negative correlation between the BMI and frequency of peripheral blood monocytes. This is in contrast to previous reports of a positive association between CD56+ monocytes and body fat, body mass index, waist circumference, C-reactive protein, triglycerides, and HbA1c in obese individuals [71]. A possible explanation for observed discrepancies is that the BMI of our participants was in the range for normal weight.

## 5. Conclusions

In the present study, we have showed that a three-week dietary protocol with a daily consumption of two fortified hen eggs (~410 mg/day *n-3* PUFAs) had a negligible effect on the serum concentrations of *n-3* and *n-6* PUFAs, and had no effect on the skin microvascular reactivity. While this provides additional evidences for the safety of hen eggs consumption in terms of atherogenic effects and cardiovascular risk, results of this study also suggested that the minimal dose of *n-3* PUFAs in the dietary protocols aiming to improve microvascular and endothelial function should be higher. Based on our recently published studies and available literature, *n-3* PUFA dose should range from 750 mg to 1 g.

Here, we also demonstrated a small but significant immunomodulatory effect of hen egg consumption in respect to reduced integrin CD11a/LFA-1 expression on monocyte and granulocyte populations accompanied by a shift from classical to intermediate monocyte subsets. These effects were slightly more pronounced in low-dose *n-3* PUFA-fortified eggs. The proposed *n-3* PUFAs’ mediated effects on the microcirculation are summarized in Figure 3.

A limitation of this study was that the participants recruited to the *n-3* PUFAs group had slightly (not significantly) higher body mass index, which was in the normal range; however, this had resulted in significantly higher body fat composition in the *n-3* PUFAs group. The same group showed significant correlation of BMI and markers of oxidative balance, which is probably due to higher levels of lipid peroxidation in those individuals. Interestingly, consumption of low-dose *n-3* PUFA-fortified eggs resulted in increased SOD activity, which insufficiently compensated to the observed reduction of FRAP (a measure of total antioxidative capacity); however, positive association between SOD activity and the frequency of peripheral blood monocytes suggest that the increased SOD activity may compensate for increased inflammation, e.g., decrease monocyte activation and extravasation.

Nevertheless, future studies should be directed toward the functional status of the immune cells, e.g., it is possible to speculate that reduced frequencies of classical monocytes are related to a shift from primarily pro-inflammatory, ROS-mediated phagocytic response to antigen presentation, immune regulation, or antibody/complement-mediated effector function. Additionally, assessment of oxidative status of leukocyte subpopulations would be an important link between the cardioprotective effects of *n-3* PUFAs consumption and low-grade inflammation affecting endothelial function, which is observed in all cardiometabolic diseases.

## Figures and Tables

**Table 1:** Fatty acid profile of hens’ feeding mixture and edible parts of regular (consumed by controls) and *n-3* PUFA-enriched eggs (consumed by *n-3* PUFAs group).

	Feeding Mixture (*n* = 3) (g/100 g Total Fatty Acids)		Eggs (*n* = 10) (mg/100 g Egg ^1^)	
Fatty acid	For production of regular eggs	For production of *n-3* PUFA-enriched eggs	Regular eggs	*n-3* PUFA-enriched eggs
∑SFA	21 ± 0.8	17 ± 0.3 *	3107 ± 104	2192 ± 71 *
∑MUFA	27 ± 1.4 *	37 ± 1.2	4091 ± 180	2980 ± 174 *
∑*n-6* PUFA	49 ± 1.8	33 ± 1.5 *	1775 ± 270	1397 ± 153
LA	42 ± 2.5	33 ± 1.2 *	1551 ± 258	1302 ± 146
AA	n.d.	0.04 ± 0.01	197 ± 15	79 ± 10 *
∑*N-3* PUFA	3.47 ± 0.69 *	14 ± 1.88	75 ± 4 *	403 ± 49
ALA	3.33 ± 0.70 *	11.9 ± 1.97	23 ± 2.9 *	198 ± 55
EPA	n.d.	0.63 ± 0.06	12 ± 2.0 *	22 ± 3.2
DHA	0.14 ± 0.02 *	0.93 ± 0.05	39 ± 3.5 *	180 ± 14
∑*n-6*/*n-3* PUFA	14.07	2.44 *	23.79	3.46 *

Data are presented as mean ± standard deviation (SD). *n*—number of analysis; ∑SFA—saturated fatty acids (C14:0, C15:0, C16:0, C17:0, C18:0, C20:0, C21:0, C23:0); ∑MUFA—monounsaturated fatty acids (C14:1, C16:1, C18:1n9t, C18:1n9c, C20:1n9, C22:1n9); ∑*n-6* PUFA—polyunsaturated fatty acids (C18:2n6c, C18:3n6, C20:3n6, C20:4n6, C22:2n6); LA—linoleic acid (C18:2n6c); AA—arachidonic acid (C20:4n6); ∑*n-3* PUFA—polyunsaturated fatty acids (C18:3n3, C20:3n3, C20:5n3, C22:6n3); ALA—alpha linolenic acid (C18:3n3); EPA—eicosapentaenoic acid (C20:5n3); DHA—docosahexaenoic acid (C22:6n3); n.d—non-detected. ^1^ edible part; * *p* < 0.05 control vs. *N-3* PUFAs.

**Table 2:** Cardiovascular and biochemical characteristics of study population before and after consumption of regular (control group) or *n-3* PUFA-enriched hen eggs (*n-3* PUFAs group).

Parameter	Control Group		*n-3* PUFA Group	
	Before	After	Before	After
*N* (W/M)	9 (2/7)		11 (7/4)	
Age (years)	21 ± 2		21 ± 2	
SBP (mmHg)	124 ± 12	122 ± 10	117 ± 10	118 ± 8
DBP (mmHg)	78 ± 9	75 ± 10	75 ± 9	77 ± 7
MAP (mmHg)	93 ± 9	91 ± 9	89 ± 9	90 ± 6
HR (beats per minute)	75 ± 16	68 ± 11	72 ± 15	71 ± 10
erythrocytes (×10^12^/L)	4.9 ± 0.4	5.0 ± 0.5	4.8 ± 0.5	4.8 ± 0.5
hemoglobin (g/L)	144 ± 12	148 ± 14 *	139 ± 14	139 ± 13
hematocrit (%)	42.3 ± 3.8	43.9 ± 4.0 *	41.7 ± 3.8	42.2 ± 3.9
leukocytes (×10^9^/L)	7.3 ± 1.1	7.2 ± 1.6	7.5 ± 1.6	8.2 ± 1.3
thrombocytes (×10^9^/L)	231 ± 36	258 ± 39	266 ± 69	289 ± 75 *
urea (mol/L)	5.7 ± 1.4	5.5 ± 1.4	5.4 ± 1.5	5.4 ± 1.4
creatinine (µmol/L)	83 ± 15	80 ± 16	79 ± 13	79 ± 14
urates (µmol/L)	343 ± 27	332 ± 41	298 ± 76	291 ± 69
sodium (mol/L)	138 ± 2	137 ± 2	140 ± 2 †	139 ± 2
potassium (mol/L)	4.1 ± 0.2	4.1 ± 0.3	4.3 ± 0.2	4.4 ± 0.5
calcium (mol/L)	2.4 ± 0.1	2.5 ± 0.1	2.4 ± 0.1	2.5 ± 0.1
iron (µmol/L)	16 ± 5	17 ± 6	18 ± 8	15 ± 9
transferrin (g/L)	2.5 ± 0.3	2.7 ± 0.3 *	2.7 ± 0.2	3.0 ± 0.4 *
glucose (mol/L)	5.2 ± 0.6	4.9 ± 0.7	4.9 ± 0.4	4.4 ± 0.4 *
hsCRP (mg/L)	1.7 ± 2.8	0.6 ± 0.4	1.6 ± 2.3	1.2 ± 1.0
cholesterol (mol/L)	4.0 ± 0.7	4.2 ± 0.3	4.5 ± 0.8	4.6 ± 0.8
triglycerides (mol/L)	1.1 ± 0.3 †	1.1 ± 0.6	0.7 ± 0.3	0.9 ± 0.5
HDL cholesterol (mol/L)	1.4 ± 0.2	1.4 ± 0.2	1.7 ± 0.4 †	1.7 ± 0.4
LDL cholesterol (mol/L)	2.1 ± 0.5	2.3 ± 0.2	2.3 ± 0.4	2.4 ± 0.5
apoA (g/L)	1.6 ± 0.2	1.6 ± 0.2	1.8 ± 0.3	1.7 ± 0.3
apoB (g/L)	0.6 ± 0.1	0.7 ± 0.1	0.7 ± 0.1	0.7 ± 0.1

Data are presented as mean ± standard deviation (SD). *N*—number of participants; W—women; M—men; SBP—systolic blood pressure; MAP—mean arterial pressure; DBP—diastolic blood pressure; HR—heart rate; hsCRP—high-sensitivity C reactive protein; LDL—low-density lipoprotein; HDL—high-density lipoprotein; apoA—apolipoprotein A1; apoB—apolipoprotein B. * *p* < 0.05 control vs. *n-3* PUFAs; † *p* < 0.05 before vs. after within control or *n-3* PUFAs group.

**Table 3:** Anthropometric Measures, Body Fluid Status and Body Composition Responses to Regular (Control Group) or *n-3* PUFAs Enriched Hen Eggs Consumption (*n-3* PUFAs Group).

Parameter	Control Group		*n-3* PUFAs Group	
	Before	After	Before	After
BMI (kg/m^2^)	24.4 ± 2.3	24.3 ± 2.3	25.2 ± 3.7	25.0 ± 4.2
WHR	0.80 ± 0.02	0.80 ± 0.02	0.79 ± 0.04	0.77 ± 0.04
Fat Free Mass (%)	83.6 ± 7.7 *	83.0 ± 7.9	75.1 ± 7.6	75.3 ± 8.8
Fat (%)	16.4 ± 7.5	17.0 ± 7.9	24.9 ± 7.6 *	24.7 ± 8.8
Total Body Water (%)	62.9 ± 8.7	61.6 ± 7.5	56.0 ± 7.0	55.8 ± 7.6
Extracellular Water (%)	43.8 ± 2.8	43.0 ± 1.4	42.7 ± 1.5	42.8 ± 1.8
Intracellular Water (%)	56.2 ± 2.8	57.0 ± 1.4	57.3 ± 1.5	57.2 ± 1.8
Plasma Fluid (L)	4.61 ± 0.98	4.44 ± 1.07	3.79 ± 0.98	3.79 ± 1.22
Body Density (kg/L)	1.061 ± 0.015 *	1.060 ± 0.020	1.043 ± 0.017	1.043 ± 0.020

Values are mean ± SD. BMI—body mass index; WHR—waist-to-hip ratio. * *p* < 0.05 Control vs. *n-3* PUFAs.

**Table 4:** Serum fatty acid profile changes in response to regular (control group) or *n-3* PUFA-enriched hen eggs consumption (*n-3* PUFAs group).

Parameter	Control Group (*n* = 9)		Omega-3 Group (*n* = 11)	
	Before	After	Before	After
%SFA (%FAME)	36.7 ± 2.2	33.9 ± 10.2	36.9 ± 1.8	29.3 ± 11.7
%MUFA (%FAME)	23.2 ± 5.1	24.7 ± 10.0	19.4 ± 2.1	28.1 ± 14.9
%PUFA (%FAME)	40.6 ± 5.5	41.3 ± 5.5	43.6 ± 2.8	42.6 ± 4.8
SFA (%FAME)				
C14:0 Myristic acid	0.82 ± 0.74	1.31 ± 0.76	1.02 ± 0.34	1.30 ± 0.57
C16:0 Palmitic acid	25.8 ± 2.9	25.5 ± 3.3	26.6 ± 1.6	27.0 ± 2.5
C18:0 Stearic acid	10.1 ± 2.0	10.2 ± 3.8	9.5 ± 1.1	9.3 ± 1.2
MUFA and PUFA (%FAME)				
C16:1 (*n-7*) Palmitoleic acid	1.21 ± 0.76	1.49 ± 0.55	1.24 ± 0.28	1.40 ± 0.65
C18:1 (*n-7*) Vaccenic acid	1.24 ± 0.72	1.67 ± 0.31	1.29 ± 0.18	1.38 ± 0.24
C18:1 (*n-9*) Oleic acid	20.7 ± 4.6 †*	18.4 ± 2.4	16.6 ± 2.1	16.9 ± 2.8
C18:2 (*n-6*) Linoleic acid	30.3 ± 4.9	30.4 ± 4.3	34.0 ± 2.9	32.8 ± 4.1
C20:3 (*n-6*) Dihomo-gamma-linolenic acid	1.46 ± 0.73	1.51 ± 0.73	1.74 ± 0.91	1.69 ± 0.73
C20:4 (*n-6*) Arachidonic acid	7.0 ± 2.4	7.7 ± 1.6	6.8 ± 1.5	6.8 ± 1.6
C18:3 (*n-3*) α-Linolenic acid	1.04 ± 1.86 *	0.61 ± 1.1.10	0 ± 0	0.08 ± 0.18
C20:5 (*n-3*) Eicosapentaenoic acid	0 ± 0	0 ± 0	0 ± 0	0 ± 0
C22:6 (*n-3*) Docosahexaenoic acid	0.85 ± 0.86	1.14 ± 0.26	0.91 ± 0.48	1.09 ± 0.66
*n-6*/*n-3* PUFA	20.5	22.6	46.7	35.3

Results are expressed as mean ± standard deviation (SD). %FAME—fatty acid methyl esters; MUFA—monounsaturated fatty acids; SFA—saturated fatty acids; PUFA—polyunsaturated fatty acids. * *p* < 0.05 control vs. *n-3* PUFAs; † *p* < 0.05 before vs. after within control or *n-3* PUFAs group.

**Table 5:** Lipid peroxidation (TBARS and LOOH), antioxidant capacity (FRAP), and antioxidative enzyme (GPx and SOD) activity responses to regular (control group) or *n-3* PUFA-enriched hen eggs consumption (*n-3* PUFAs group).

Parameter	Control Group		*n-3* PUFAs Group	
	Before	After	Before	After
TBARS (µM MDA)	23.5 ± 0.6	24.1 ± 2.1	26.7 ± 2.6 *	26.1 ± 2.8 *
FRAP (mM Trolox)	0.47 ± 0.06	0.48 ± 0.05	0.46 ± 0.07	0.42 ± 0.08 †
GPx (U/mg protein)	3.83 × 10^−3^ ± 0.7 × 10^−3^	3.45 × 10^−3^ ± 0.5 × 10^−3^	3.59 × 10^−3^ ± 0.4 × 10^−3^	4.04 × 10^−3^ ± 0.9 × 10^−3^
SOD (U/mg protein)	0.11 ± 0.05	0.10 ± 0.02	0.06 ± 0.06	0.10 ± 0.10 †
LOOH (µM)	1.46 ± 0.66	1.35 ± 0.51	1.30 ± 0.65	1.10 ± 0.47

FRAP—Ferric reducing ability of plasma; TBARS—thiobarbituric acid reactive substances; SOD—superoxide dismutase; GPx—glutathione peroxidase; LOOH—lipid hydroperoxide. * *p* < 0.05 control vs. *n-3* PUFAs (*t* test); † *p* < 0.05 before vs. after within control or *n-3* PUFAs group (one-way ANOVA).

**Table 6:** Body mass index correlates to frequencies of peripheral blood monocytes and markers of oxidative balance.

	Control Group		*n-3* PUFAs Group	
	Body Mass Index (kg/m^2^)			
	***r* =**	***p =***	***r* =**	***p =***
Rate of peripheral blood monocytes (%)	−0.684	0.010 *	−0.468	0.039 *
FRAP (mM Trolox)	0.092	0.717	0.534	0.015 *
TBARS (µM MDA)	0.113	0.654	0.462	0.039 *
SOD activity (U/mg protein)	−0.052	0.839	−0.548	0.028 *
GPx activity (U/mg protein)	−0.066	0.793	−0.376	0.146
LOOH (µM)	−0.252	0.313	−0.098	0.665

Pearson correlation coefficient was calculated, * *p* < 0.05 was considered significant. FRAP—Ferric reducing ability of plasma; TBARS—thiobarbituric acid reactive substances; SOD—superoxide dismutase; GPx—glutathione peroxidase; LOOH—lipid hydroperoxide.

## Supplementary Materials

The following are available online at https://www.mdpi.com/2072-6643/12/10/3122/s1, Figure S1: Regular and *n-3* PUFA Enriched Eggs Consumption had no significant effect on the skin microvascular reactivity in young healthy individuals, Table S1: Frequency of CD11a+ leukocyte subpopulations, Table S2. Absolute cell number of CD11a+ leukocyte subpopulations.
