# Supplementary material for: Leukocyte Activation and Antioxidative Defense Are Interrelated and Moderately Modified by n-3 Polyunsaturated Fatty Acid-Enriched Eggs Consumption—Double-Blind Controlled Randomized Clinical Study
Source: Nutrients. 2020 Oct 13;12(10):3122. doi: 10.3390/nu12103122 (PMC7650765; doi:10.3390/nu12103122)
Supplement: Supplementary file 1 [file nutrients-12-03122-s001.zip › Supplementary material - revised.docx]

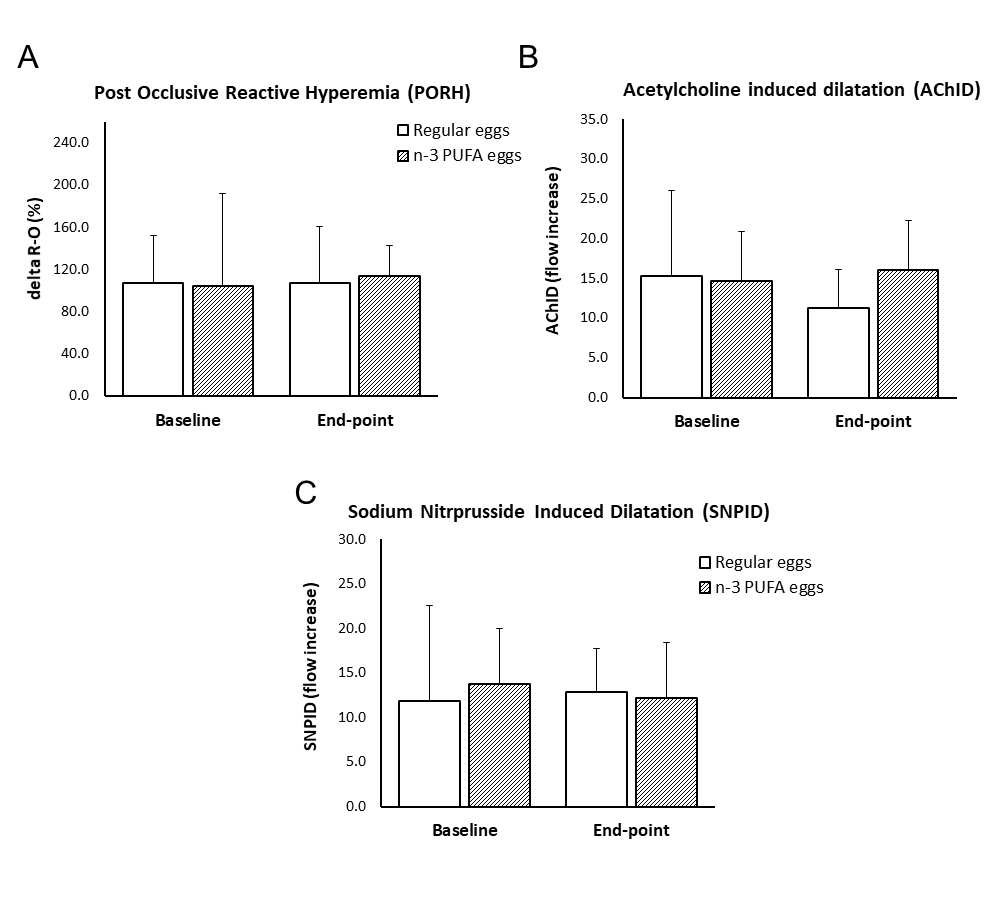


**Supplemental Figure 1. Regular and n-3 PUFA Enriched Eggs Consumption had no significant effect on the skin microvascular reactivity in young healthy individuals.**

(A) Post-occlusive reactive hyperemia (PORH), (B) Acetylcholine-induced dilation (AChID), and (C) Sodium nitroprusside induced dilation (SNPID), PORH measurement is expressed as the difference between percentage of flow change during reperfusion and occlusion in relation with baseline (R-O%). AChID and SNPID are expressed as flow increase following Ach or SNP administration compared to baseline flow. Data are presented as average ± SD. Regular eggs – group of young healthy volunteers subjected to 14-day diet with regular eggs; n-3PUFA eggs – group of young healthy volunteers subjected to 14-day diet with n-3 PUFA enriched eggs.

| **Supplemental table 1. Frequency of CD11a+ leukocyte subpopulations** | | | | |
| --- | --- | --- | --- | --- |
|  | Control | | n-3 PUFAs | |
|  | Before | After | Before | After |
| N *(W/M)* | 9 (2/7) | | 11 (7/4) | |
| Granulocytes  *(% of total CD45+ leukocytes)* | 64.8±8.16 | 69.3±4.43 | 68.0±8.70 | 64.3±14.4 |
| Monocytes  *(% of total CD45+ leukocytes)* | 3.35±1.02 | 3.53±1.49 | 4.10±1.93 | 3.91±1.30 |
| Lymphocytes  *(% of total CD45+ leukocytes)* | 15.5±7.16 | 16.1±5.79 | 15.9±8.83 | 18.6±6.13 |
| *Data are presented as mean±SD. Within group differences were teste using paired t-test. while between group differences were tested by one way ANOVA. p<0.05 was considered significant.* | | | | |

| **Supplemental table 2. Absolute cell number of CD11a+ leukocyte subpopulations** | | | | |
| --- | --- | --- | --- | --- |
|  | Number of CD11a+ granulocytes (x10e9/L) | | | |
|  | Control | | n-3 PUFAs | |
|  | After | Before | After | 2. mjerenje |
| N *(W/M)* | 9 (2/7) | | 11 (7/4) | |
| Granulocytes  *(x10e9/L)* | 4.27±1.67 | 5.01±1.24 | 5.29±1.52 | 5.28±1.75 |
| Monocytes  *(x10e9/L)* | 0.22±0.11 | 0.24±0.09 | 0.31±0.14 | 0.31±0.09 |
| Lymphocytes *(x10e9/L)* | 1.05±0.73 | 1.12±0.38 | 1.08±0.50 | 1.51±0.42 |
| *Data are presented as mean±SD. Within group differences were teste using paired t-test. while between group differences were tested by one way ANOVA. p<0.05 was considered significant.*  *The cell numbers were calculated based on total leukocytes numbers given in differential blood count.* | | | | |
