# Supplementary figures and images for: Leukocyte Activation and Antioxidative Defense Are Interrelated and Moderately Modified by n-3 Polyunsaturated Fatty Acid-Enriched Eggs Consumption—Double-Blind Controlled Randomized Clinical Study
Source: Nutrients. 2020 Oct 13;12(10):3122. doi: 10.3390/nu12103122 (PMC7650765; doi:10.3390/nu12103122)

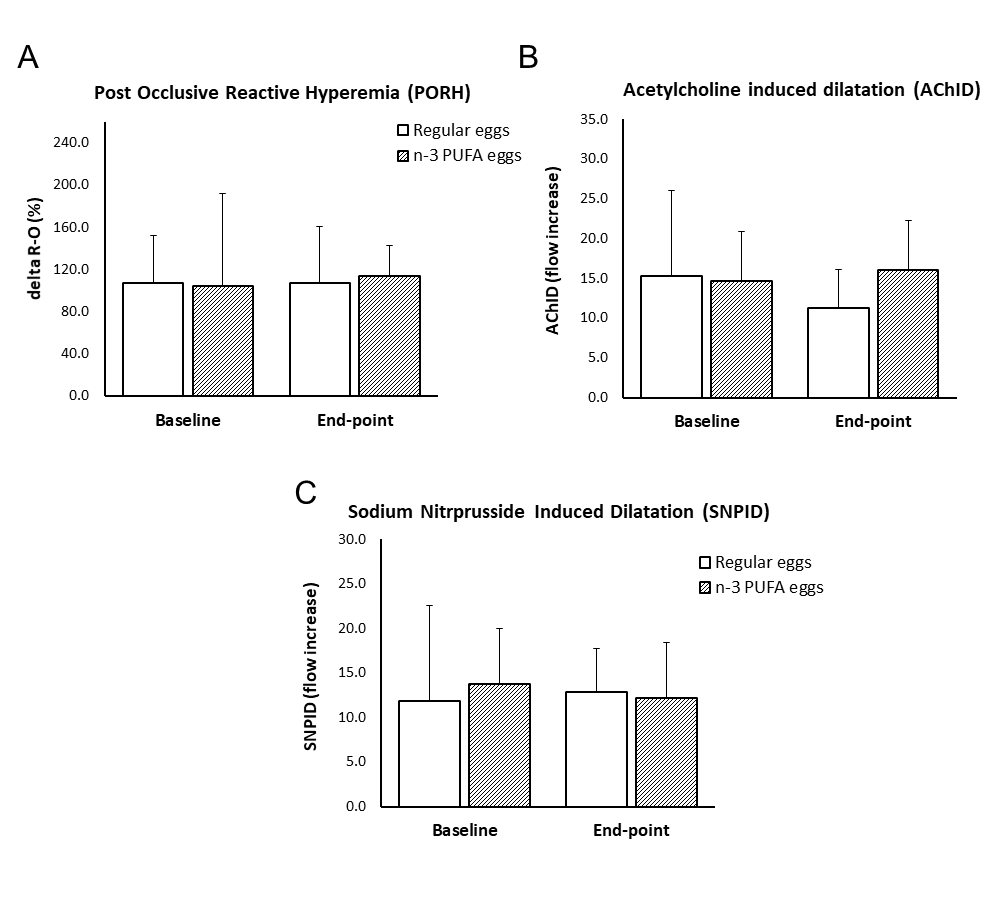

Supplement: Supplementary file 1 [file nutrients-12-03122-s001.zip › Supplementary Fig 1.tif]
